# To switch or not to switch: Intentions to switch to injectable PrEP among gay and bisexual men with at least twelve months oral PrEP experience

**DOI:** 10.1371/journal.pone.0200296

**Published:** 2018-07-19

**Authors:** Kathrine Meyers, Yumeng Wu, Atrina Brill, Theodorus Sandfort, Sarit A. Golub

**Affiliations:** 1 Aaron Diamond AIDS Research Center, The Rockefeller University, New York, New York, United States of America; 2 Department of Psychology, Hunter College of the City University of New York, New York, New York, United States of America; 3 HIV Center for Clinical and Behavioral Studies, Columbia University, New York, New York, United States of America; 4 Department of Psychology, the Graduate Center of the City University of New York, New York, New York, United States of America; Centers for Disease Control and Prevention, UNITED STATES

## Abstract

**Background:**

Phase III trials of long-acting injectable (LAI) PrEP, currently underway, have great potential for expanding the menu of HIV prevention options. Imagining a future in which multiple PrEP modalities are available to potential users of biomedical HIV prevention, we investigated which factors might help direct a patient-physician shared-decision making process to optimize the choice of biomedical HIV prevention method.

**Methods:**

Participants (n = 105; ages 19–63; 46.7% men of color) were former participants in a PrEP demonstration project and had taken daily oral PrEP for ≥ 12 months. Participants were given information about LAI PrEP and asked whether they would be interested in switching from oral to LAI PrEP. Participants were also asked about specific pros/cons of LAI PrEP, PrEP attitudes and experiences, and personality factors.

**Results:**

Two-thirds (66.7%) of current oral PrEP users would switch to LAI PrEP. Intention to switch was associated with product-level and psychosocial factors. Attitudes towards logistical factors (i.e. getting to regular clinic visits for recurring shots) featured more prominently than factors related to the physical experience of PrEP modality (i.e., concerns about injection pain) as motivators for switching. In a multivariate regression model, psychosocial factors including the emotional burden of daily pill taking, deriving a sense of responsibility from PrEP use, and self-identifying as an early adopter, were the strongest predictors of switching.

**Conclusions:**

These data underscore the importance of attending not only to product-level factors, but also to the logistical and psychological experience of prevention methods for users. Findings have significant implications for the development of patient education materials and patient-provider shared decision aids.

## Introduction

Tenofovir-based oral PrEP is the only biomedical approach currently approved to prevent HIV. Other ARV-based prevention modalities—including vaginal rings, microbicides, alternate oral agents, injectables—are currently in clinical development, with the long-acting integrase inhibitor Cabotegravir Long-Acting (Cab-LA) furthest along. With the prevention efficacy of Cab-LA under investigation in a global study of men who have sex with men (MSM) and transgender women who have sex with MSM [1], a series of sociobehavioral studies have begun assessing attitudes towards long-acting injectable PrEP (LAI-PrEP) among gay men in the US and other settings [1–9]. These studies show variable enthusiasm for an injectable PrEP product, with positive attitudes towards LAI PrEP (as measured by acceptability, preference or willingness to use) ranging from 25.2% [3] to 80.0% [6]. This variability is likely due to heterogeneity in study design, wording of survey questions, different explanation of LAI-PrEP, and/or real differences between populations. However, taken together, these studies do suggest that the addition of a second PrEP modality would likely attract PrEP-naïve users for whom the daily oral pill was not acceptable and consequently increase overall PrEP use [9]. While useful in establishing acceptability of attributes of a particular product-type at the most basic level, clinical trials are limited in the depth with which they can collect data on the acceptability of a specific biomedical prevention technology among potential users.

For this new biomedical prevention strategy to be effective, it is important to understand not only interest in Cab-LA during clinical development, but also its likelihood of being adopted by real users and the factors that might underlie decisions about uptake. People who are already on oral PrEP present a particularly informative population in which to study this question, as these individuals have experienced the physical and psychosocial benefits and challenges of daily oral PrEP use first-hand. For example, they may have experienced a decrease in anxiety around sex [10, 11], an increase in intimacy with partners [10, 12], an increase in sexual satisfaction [10, 13], worry about missing doses [14, 15], changes in frequency of condomless sex [10, 16, 17], infection with an STI [18], or increases in stigma related to sexual practices and choices in HIV prevention methods [13, 19]. In addition, current oral PrEP users understand the logistics of taking PrEP, including regular clinic visits [20], timely pharmacy refills [21], storing of pills [22, 23], and integrating the medication into their routines and lifestyles [22, 23]. The experience of integrating oral PrEP into their daily lives allows them to provide answers to questions about a hypothetical new injectable formulation of PrEP, informed by real world practice. Some of these experiences may continue to exist with the use of an injectable product, some may be overcome by its development, and some distinct new experiences are likely to arise as well.

The goal of this study was to investigate specific factors that might help direct a patient-provider shared decision-making process to optimize the choice of biomedical HIV prevention method. Such tools have been utilized in the contraceptive field [24–27] and our objective was to generate preliminary data that could usefully differentiate between current oral PrEP users who would or would not switch to LAI-PrEP. Specifically, the study focused on product-related and psychosocial factors that may impact implementation of LAI-PrEP. We tested measures that we hypothesized would help us understand who would be a good candidate for an intra-muscularly-injected versus an orally-administered PrEP product. Hypothesized domains included: 1) product-related factors, such as the physical experience of the PrEP modality and logistical features of administration; and 2) psychosocial factors, like agency and control, psychosocial aspects of adherence, and self-identification as an early-adopter or risk taker.

## Materials and methods

### Study population and procedures

Participants were recruited from among former participants of a PrEP demonstration project in New York City (NIH RO1: MH106380). Eligible participants were assigned male sex at birth, over age 18, had been prescribed PrEP in the context of the SPARK PrEP Demonstration Project, and reported having taken PrEP for at least 12-months. All eligible participants of the original study who had indicated interest in being contacted for future research participation were contacted by research staff (n = 237). Of these, 72 (27%) never responded to our contact, 27 (11%) declined to participate, 32 (12%) scheduled a study visit but did not attend, and 106 (40%) completed a study visit. Those who completed study visits did not differ significantly from those in the other three groups on age, race/ethnicity, education, income, or insurance status. One participant left the majority of his survey blank, and was excluded from further analysis, leaving a final analytic sample of 105.

Participants completed a Qualtrics-programmed survey on a computer during an in-person visit at Hunter College and received $40 compensation upon completion. All participants completed an informed consent process with study staff and signed a written consent form. The study was reviewed and approved by the Human Research Protections Program at the City University of New York (IRB file number 2015–0455).

### Survey design and measurement

Participants were given the following written description of the “PrEP shot”:

“an anti-HIV medication that is made into an injection (shot) that can remain in the blood for a long period of time. You take one month of daily pills to make sure you don’t have a reaction to the medication. Then come to a clinic every three months to get two shots in the buttocks by a medical provider.”

Following this description, participants completed self-report survey items. The survey instrument included a series of close-ended and open-ended questions developed specifically for this study, based on the research team’s experience investigating the use of daily oral PrEP and injectable PrEP among users [28, 29] and clinical trial participants [30]. Because these measures were developed for this project, and because few (if any) acceptability measures exist specific to LAI PrEP, we have provided our complete list of measures in Table 1. We used four types of items and response choice sets to measure participant attitudes, including: a) pro/con judgements, in which participants read characteristics and were asked to rate the extent to which each was a “pro” or a “con” of the shot compared to the PrEP pill; b) Polar or “A versus B” items, in which participants are presented with two opposite statement and were asked to rate themselves between them; c) Likert-type scale items, in which participants responded to statements about injections and pills on a seven-point scale ranging from “very challenging” to “very easy”; and d) classic Likert-scale items, in which participants responded to statements on a seven-point scale ranging from “strongly agree” to “strongly disagree”. As demonstrated in Table 1, measures were divided into “product-level” factors, including physical experience and logistics/convenience and “psychosocial factors’, which included feelings of control, psychosocial attitudes/preferences, and feelings about adherence.

**Table 1:** Hypothesized predictors of LAI PrEP switch intentions.

Concepts	Indicators	Measures		
A. *Pro/Con Judgments*. Participants indicate the extent to which each is a pro or a con of the PrEP shot on a 7-point scale, ranging from 1 (this is a pro of the shot) to 7 (this is a con of the shot).				
Physical Experience	Peri-injection pain	I would be worried about pain during the shots		
	Injection blemish	I would be worried that there would be a bump or a mark from the shots.		
	Post-injection pain	I would be worried about pain in the days after the shot.		
Logistics/Convenience	Injection interval	I would have to get two shots in the butt every 8–12 weeks.		
	Daily pill-taking	I wouldn’t need to take a pill every day.		
	Adherence when partying	I wouldn’t have to worry about remembering to take medications when I party.		
	Adherence when traveling	I wouldn’t have to remember to bring pills with me when I sleep somewhere new or go on a trip.		
Feeling of Control	Logistical control	I wouldn’t have control over starting and stopping the medication because it would be in my body for 8–12 weeks after the shots.		
	Psychological control	I would feel less in control of my HIV prevention because I’m not actively taking a pill daily		
	Reliability concerns	I would be worried that the shot would stop working in my body and I wouldn’t know.		
*B*. *Polar items (A vs*. *B) items*. Participants place themselves along a 7-point line, as described below.				
Physical Experience	Pain tolerance	“I have a really low tolerance for pain or discomfort; feeling bad can really ruin my day”	1 |---| 7	“I have a high tolerance for pain or discomfort; if I feel bad, I just ignore it”
	Injection tolerance	“I hate getting shots and try to avoid them whenever possible”	1 |---| 7	“Shots don’t bother me if I need them”
	Pill aversion	“I hate swallowing pills”	1 |---| 7	“I don’t mind swallowing pills”
Psychosocial factors	Spontaneity	“I’m a ‘creature of habit,’”	1 |---| 7	“I like to be as spontaneous as possible”
	Routine preference	“I like to have a set routine that I follow every day”	1 |---| 7	“I hate having a set routine; I like to be flexible”
	Active prevention	“I like to know that I’m doing something active to control my HIV risk”	1 |---| 7	“I like having an HIV prevention strategy that I never have to think about”
	Protection consistency	“I like an HIV prevention method that is active in my body all the time so I’m always protected”	1 |---| 7	“I like an HIV prevention method that I use only when I actually need it”
	Early Adopter	“I’m the kind of person who is always the first to try something new”	1 |---| 7	“I’m the kind of person who is slow to try new things”
*C*. *Likert-scale judgments ranging from 1 (strongly disagree) to 7 (strongly agree)*				
Feelings about adherence	Emotional burden of daily pills	Taking PrEP pills every day feels like an emotional burden		
	Personal responsibility	Taking PrEP pills every day makes me feel responsible		
*D*. *Likert-type scale judgments*, *ranging from 1 (very challenging) to 7 (very easy)*				
Logistics/Convenience	Pill pick-up	Picking up refills of PrEP pills		
	Shot schedule	Returning to the clinic every 8–12 weeks to get your shots		
	Pill frequency	Remembering to take a pill every day		
	Clinic visit interval	Coming to the clinic for visits every 3 months		

#### Willingness to switch

After completing these survey measures, participants were asked: “If the PrEP shot were FDA approved and available for the same cost as the daily PrEP pill, how likely would you be to switch to the PrEP shot?” on a 5-point scale from 1, definitely switch to 5, definitely not switch with mid-point “unsure”. Those who would definitely or probably switch were categorized as “switchers”, those who were unsure, or would probably or definitely not switch were categorized as “non-switchers”.

#### Demographic variables

As part of the survey, participants were asked to report their age, race/ethnicity, sexual orientation, education, employment, and income. Additional items collected information on living situation and partnership characteristics.

We also asked three open-ended questions to elicit more details on participants’ perspectives on the relative pros and cons of the PrEP shot as compared to daily oral PrEP: (1) What do you think is the biggest benefit or “pro” of the PrEP shot compared to the PrEP pill?; (2) What do you think is the biggest drawback or “con” of the PrEP shot compared to the PrEP pill?; (3) Please tell us a little bit about why you would or wouldn’t switch.

We included open-ended questions for several reasons. First, given the preliminary nature of the constructs and measures we developed to capture them, we wanted participants to be able to raise issues that we may not have previously considered and provide more depth to their survey responses [31, 32]. Second, we anticipated that some items might lack variability in responses due to the potential homogeneity in a cohort of early oral PrEP adopters. Such a lack of variability may impact on the quantitative analysis and significance of findings; therefore, we included open-ended fields that would allow us to pick up common themes.

Across the three open questions, 97.5% of respondents provided answers. Text entered by participants in response to the three open-ended questions was organized thematically around the motivators and barriers to switching identified in the statistical analysis. One researcher coded and categorized the data. Two other researchers reviewed the coding and final coding was based on group discussion. Excerpts were chosen to provide a more detailed explanation of the respondents’ answers to the survey items.

### Statistical analysis

We first examined bivariate associations between the outcome (i.e., intention to switch) and each of the predictor variables. Variables that were significant in bivariate testing (p < .10) were entered into a multivariable logistic regression model. We report both bivariate and adjusted odds ratios. All continuous measures were z-scored for ease of comparison across measures with different scales; increases in odds are associated with standard deviation increases in any given measure. Analysis was performed in SPSS 24.0.

## Results

### Intention to switch from oral to LAI-PrEP

The sample was largely gay (89.5%), ethnically diverse (46.7% non-white), and fairly well distributed in terms of education, income, and insurance status (Table 2). Almost 92% of the sample (n = 96) were still taking PrEP. Two-thirds of the sample reported that they would definitely (36.2%) or probably (30.5%) switch from daily oral PrEP to LAI-PrEP. About one fifth (21.0%) were unsure whether or not they would switch, a tenth (9.5%) would probably not switch, and 3 out of 106 respondents (2.9%) would definitely not switch.

**Table 2:** Sample characteristics (n = 105).

Demographics	n (%)
**Age**	
<30	40 (38.1)
> = 30	65 (61.9)
**Race and ethnicity**	
Black	10 (9.5)
Latino/Hispanic	28 (26.7)
White	56 (53.3)
Multiracial	6 (5.7)
Other	5 (4.8)
**Sexual orientation**	
Gay	94 (89.5)
Bisexual	7 (6.7)
Queer	4 (3.8)
**Education**	
Less than 4-year college degree	33 (31.4)
4-year college degree or more	72 (68.6)
**Employment**	
Full-time	62 (59.0)
Not full-time	43 (41.0)
**Annual income**	
<$30,000	36 (34.3)
$30,000-$50,000	38 (36.2)
>$50,000	31 (29.5)
**Insurance Status**	
Private Insurance	52(49.5)
Medicaid	25 (23.8)
Uninsured	21 (20.0)
Living status	
**Living alone**	34 (32.4)
Not living alone	71 (67.6)
**Do you have a partner at this time?**	
I am in a committed relationship	26 (24.8)
I have a boyfriend or a girlfriend	16 (15.2)
I am casually dating	21 (20.0)
I am single	42 (40.0)
**Main partner on PrEP (n = 42)**	
Yes	15 (35.7)
No	27 (64.3)
**Sexual relationship with main partner (n = 42)**	
Neither of us has sex with others, we are monogamous	14 (33.3)
We both have sex with others	21 (50.0)
Only I have sex with others	4 (9.5)
I have sex with others; I don’t know what my partner does	2 (4.8)
I don’t have sex with others; I don’t know what my partners does.	1 (2.4)
**Current PrEP User**	96 (91.4)

### Factors associated with intention to switch

Intention to switch to LAI from oral PrEP was not associated with age, race/ethnicity, sexual orientation, education, insurance status, factors related to partnership type, or whether or not the participant was a current PrEP user. However, income was associated with intention to switch; specifically those with moderate income ($30,000-$50,000) were more likely to consider switching, compared to those who reported highest income (Table 3). Income was included in the final model.

**Table 3:** Factors associated with intention to switch from oral to injectable PrEP (n = 105).

	OR	95% C.I.	aOR	95% C.I.
**Product level factors**				
**Physical experience**				
Injection tolerance	1.64*	(1.09, 2.47)	0.75	(0.30, 1.89)
Concerns about post-injection pain	0.57*	(0.37, 0.90)	0.81	(0.36, 1.80)
**Logistical/convenience**				
Injection interval is a con of the shots	0.30***	(0.17, 0.54)	0.37*	**(0.15, 0.91)**
Clinic visits for injections are difficult	0.37***	(0.22, 0.60)	0.31*	**(0.11, 0.91)**
**Psychosocial level factors**				
**Psychosocial aspects of adherence**				
Taking pills daily is an emotional burden	1.54†	(0.92, 2.59)	13.71**	**(2.05, 91.86)**
**Agency/control**				
Taking PrEP pills every day makes me feel responsible	1.87**	(1.20, 2.92)	4.72**	**(1.60, 13.96)**
Not taking a pill every day would make me feel less in control of my HIV prevention	0.55*	(0.35, 0.88)	0.42	(0.17,1.03)
I wouldn’t have control over starting and stopping the medication	0.56*	(0.36, 0.88)	0.83	(0.35, 1.93)
**Early adopter**				
Early adopter	2.88***	(1.73, 4.81)	3.77**	**(1.37, 10.35)**
**Demographics**				
**Income**				
Less than $30,000	2.42	(0.89, 6.59)	4.07	(0.63,26.11)
$30,000-$50,000	4.00*	**(1.40,11.44)**	5.62*	**(1.01, 31.33)**
More than $50,000	ref		ref	

Note.

^†^: p = .10

*: p<0.05,

**: p<0.01,

***: p < .001.

In bivariate analysis, intention to switch to LAI-PrEP was positively associated (p < .10) with four product-level factors and five psychosocial factors (Table 3). Analysis of the qualitative data allowed us to include illustrative quotes for the bivariate factors.

### Product-level motivators and barriers to switching

#### Physical experience of product use

Among six items measuring aspects of the physical experience of product use, four items did not show any association with the outcome: worries about pain during the shots, general tolerance for pain or discomfort, worries about visible marks on butt following the shot, and attitudes towards swallowing pills. In bivariate analysis, respondents who self-identified as having high injection tolerance (i.e., not being bothered by shots) had higher odds of switching (OR = 1.64), whereas those who were more concerned about post-injection pain had lower odds of switching (OR = 0.57).

Among non-switchers, a negative “gut reaction” to needles was a common theme in the qualitative responses, as captured in the following statements relating to needles and injection pain: “Although I think it’s a nice option to choose between one or the other, I personally hate needles!” (Hispanic, 25 years old, unsure). A 24-year-old Black participant reasoned that “the fact that it’s likely a large needle that causes pain in pretty much a majority of patients that lasts a fair bit of time…” made him unsure whether or not he would switch.

#### Logistics of product use

Items related to logistics of daily pill taking, like returning to the clinic for checkups and refilling prescriptions at pharmacies were not associated with switching intentions. The two significant items in bivariate analysis were around the logistics of the injections. Participants who felt more strongly that the injection interval (i.e., “I would have to get two shots in the butt every 8–12 weeks”) was a disadvantage of the shot had lower odds of intending to switch (OR = 0.30). The more challenging that someone rated the shot schedule (i.e., returning to the clinic every eight to twelve weeks), the lower their odds of switching (OR = 0.37). For example, in the words of one respondent, “Biggest drawback is that it’s a shot and that I have to actually visit a healthcare provider to receive the dosage” (Black, 29 years old, probably not switch).

In contrast, the following quote from the open-ended responses, illustrative of several similar quotes by definite switchers, elaborates on the idea that oral PrEP requires regular clinic visits anyway, so that regular visits for injections were not an additional burden: “Part of the routine of PrEP is blood work every 3 months, so a PrEP shot would fit nicely into this routine anyway, plus alleviate any need to refill and take daily pills each month” (White, 32 year-old, definitely switch). Others noted: “I don’t like to carry pills with me daily—its easy to forget, especially when you lead an incredibly busy schedule” (Hispanic, 26 years old, definitely switch). “(The shot) would make life so much easier not having to deal with pharmacies and refills” (White, 26 years old, definitely switch).

### Psychosocial motivators and barriers to switching

#### Psychological experience of adherence

Five out of six items that sought to measure the psychological aspects of adhering to a daily oral pill or returning for regular clinic visits did not show any association with intention to switch. However, despite the fact that items that queried anxiety around missed doses did not show any associations, this theme was common in the open-ended responses. As a 39-year-old multiracial definite switcher expressed: “I’d prefer [the shot] to having to take a pill everyday and worrying about missing a dose. It would be less stressful.”

Stronger agreement with the statement “taking PrEP pills every day feels like an emotional burden” was associated with intention to switch (OR = 1.54). One illustrative quote captures this notion of pill-taking as burdensome, particular when frequency of sex is inconsistent across time:

I would probably switch. My sexual activity is sporadic, I am more focused and diligent with taking my PrEP pills when I am sexually active regardless of if the behavior is protected or unprotected. Sometimes I go for months without having sex or what I believe is risky sexual exposure. PrEP seems to be a burden in the months I am not active. To take the injection and not worry about it for months would be an ideal scenario for me, provided I am truly confident the shot works. (Black, 53 years old, probably switch)

#### Agency and control

Assigning a higher score to the statement “Taking PrEP pills every day makes me feel responsible” (OR = 1.87) was associated with higher odds of switching. This was elaborated on in the following excerpts from responses to open-ended questions: “[PrEP] gives you a little more confidence because mistakes happen and you have sex with someone that you ask afterwards what their status is and knowing you aren’t just relying on them and are taking responsibility is a good thing,” (White, 26 years old, definitely switch).

Higher scores on the statement “Not taking a pill every day would make me feel less in control of my HIV prevention” were associated with lower odds of switching (OR = 0.55). The qualitative data elaborates on this sentiment in the following excerpt: “I also like actively taking a pill everyday as it makes me feel more in control. I feel like I am affirming my desire to remain HIV-free on a daily basis. Taking a shot and then forgetting about it feels sort of passive,” (Hispanic, gay, 49 years old, probably not switch). A second respondent, noted with regards to the shot: there is a “loss of control. With the pill I know I’m actively doing something every day to protect my health. There’s much less control with a shot administered by a doctor,” (White, 36 years old, definitely not switch).

The item that queried participants’ concern about waning protection across the injection interval was not significant. The concern came through in several participants’ responses to the open-ended questions. One participant articulated this with the following set of questions: “How do we know that the 3-month injection won’t be processed out of my body at a faster rate than the average person, thereby leaving me more vulnerable to seroconversion? What happens to the concentration of the medication in my body as time progresses? Do I have the same level of protection against HIV on day 90 as I do on day 1 after the shot?” (Black, 32 years old, unsure). Another noted: “I’m concerned [what] if the medicine levels will drop without notice, leaving me unprotected,” (Hispanic, 25 years old, unsure). A separate measure asking about concern of not having control over starting and stopping the medication specifically due to the long duration of protection was significant (OR = 0.56).

#### Early versus not early adopters

Those who rated themselves as “the kind of person who is always first to try new something new” had higher odds of switching, compared to people who considered themselves “slow to try new things” (OR = 2.88). “I would not want to be one of the first people to take it. I’d want to know others who have been on it for a while. The lack of long-term data is especially concerning,” (Hispanic, gay, 49 years old, probably not switch). As expressed by one 26-year-old White participant (probably not switch), “I would switch only after the long-term side effects were known. I don’t want to turn 60 and find out that I have some rare disease because of a shot I was taking to make myself healthier years ago.”

### Multivariable logistic regression model

In multivariable analysis, five predictors of intentions to switch were retained (Table 3). Two negatively predicted switch intentions: negative ratings of product-level factors (injection interval (aOR = 0.37) and shot schedule (aOR = 0.31)). Three psychosocial level factors positively predicted switch intentions: identifying daily pill-taking as emotionally burdensome (aOR = 13.71), believing that PrEP signifies personal responsibility (aOR = 4.72), and self-identifying as an early adopter (aOR = 3.77).

## Discussion

This is the first study to directly ask current oral PrEP users whether they would switch to an LAI-PrEP product, were it to become available, and probe the product-level and psychosocial aspects of their switch intentions. As such, these experienced PrEP users offer a unique perspective from which to assess the pros and cons of LAI-PrEP compared to daily oral PrEP. These men were among the earliest adopters of daily oral PrEP—enrolling into a PrEP demonstration within the first 24 months after FDA approval of daily oral Truvada. This makes the beliefs and attitudes of this group of men particularly relevant to the evaluation of the pros and cons of a new PrEP-modality, offering perspectives that are informed by the physical and psychological experience of oral PrEP use.

We developed twenty-four measures in five domains that we hypothesized would allow us to differentiate current oral PrEP users who would be motivated to switch to injectable PrEP from those who were likely to continue using daily oral PrEP. Measures in all five domains showed bivariate associations and four domains retained significance in the multivariable analysis.

Of the two product-level domains, physical experience was not, surprisingly, associated with switching intentions. Attitudes towards pain, injections, or pill swallowing did not differentiate those who had higher odds of intending to switch from those who were satisfied with oral PrEP in the multivariable model. However, this may be because none of our quantitative measures queried attitudes towards needles specifically, using instead, language of “shots” and “injections.” The literature on aversion to injections and needle phobia distinguishes between psychological concerns (fear of fainting, disgust), vasovagal concerns (fainting, loss of consciousness), and needle-phobia (avoidance, distress, fear) [33, 34]. This study appears to bear out this distinction as items eliciting reactions to injections were not associated with the outcome, whereas many non-switchers mentioned needles as a barrier to LAI-PrEP in their open-ended responses. Future research could use standardized scales to measure aversion to injections as compared to needles to assess whether scores on such scales could be useful in identifying someone as a candidate for injectable PrEP. These data suggest that while such concerns may not *determine* patient preferences, they should be discussed by providers as a critical component of a patient’s decision-making process.

Logistical concerns mattered for participants; concerns about the frequency of injections and clinic visits were both negatively associated with intentions to switch, whereas individuals who were not bothered by these return visits were more likely to report wanting to switch. Prior experiences during clinic visits for daily oral PrEP or other interactions with the health system may predispose individuals positively or negatively towards more frequent visits for an injectable agent. When working with patients around decision-making, providers should keep in mind that such logistical concerns may have farther-reaching influences on patients’ daily lives, and therefore may be more important than product-specific product factors such as concerns about injections. Deepening our understanding of what is difficult about clinic visits for injections—the frequency of visits, the lack of weekend and night-time hours at clinics, or the intimacy of such injections—would help planning for future implementation of LAI-PrEP as well as clinical development of future biomedical HIV prevention products.

Psychosocial-level factors also appeared to be quite important in people’s attitudes towards LAI-PrEP. The “pro” of not needing to take a daily oral pill *(i*.*e*., *adherence)* has been noted as an advantage of an LAI-PrEP modality [3, 6, 7]. However, in our data, endorsement of the statement “not having to take a pill every day” was not significantly associated with intention to switch, while the item “taking PrEP pills every day feels like an emotional burden” was. The latter item captures the psychological aspect underlying the “adherence” theme—that taking a pill every day, while doable, does take an emotional toll that seems to be operating in two ways: for some, the act of pill-taking appears to be a reminder of the risk of HIV; for others, it highlights the absence of sex. While the psychological burden of pill-taking has not been previously reported in the PrEP literature, it has been measured in the context of ARV and ARV adherence [35–37]. LAI-PrEP offers an alternative that avoids the daily psychological burden of PrEP use. Providers working with patients on decision-making may want to acknowledge the emotional burden of pill-taking as a factor in evaluation of prevention options.

Under the theme of agency, or control, the idea that taking PrEP made users “feel responsible” was most strongly associated with switching in the multivariable model. Factors related to a loss of control when switching from pill-taking to injectable PrEP were associated at the bivariate level only. Mental health benefits arising from an enhanced sense of self-efficacy and agency among oral PrEP users have been reported elsewhere [38–44], and is conceptually related to this finding. It is possible that patients who felt most affirmed by their oral PrEP use were also those who were most open to trying a new approach. Focusing on taking responsibility and being in control of HIV prevention may be an important motivating factor for providers to discuss with their patients regardless of the specific modality chosen for PrEP. It is also interesting to contrast this focus on agency with the above findings around the emotional burden of pill-taking. It may be that the ways in which individuals understand and make meaning from their PrEP behavior will be the most important factor in PrEP uptake, adherence, and persistence across modalities.

### Limitations

This study was conducted among early adopters of daily oral PrEP, which limits the generalizability of these findings to oral PrEP-experienced users. Those without experience with PrEP may respond differently. The sample was limited to cisgender men, and more data are sorely needed about experiences of PrEP use and PrEP preferences among cisgender women, and transgender and non-binary individuals. It is important to note that we asked participants to assume that insurance and financial costs of injectable PrEP would be equivalent to those they were currently facing with daily oral PrEP; however, this may not be the case during implementation. Financial constraints may be a critical factor in determining choices among PrEP modalities, and these should be considered in future implementation studies. In this sample, moderate income was most strongly associated with intentions to switch; this may be a random artifact of this particular sample, as early adoption of novel health care products and treatment is usually associated with higher income [45–46]. Another limitation is the nature of the qualitative data, which was derived not through interviews in which themes could be elaborated through a skilled interviewer, but written responses to open-ended questions. Finally, the wide confidence intervals around significant predictors signals the need for additional studies to replicate these results and assess how meaningful these factors are across different populations.

## Conclusion

Psychosocial factors appear to motivate intentions to switch to injectable PrEP among experienced PrEP users. In anticipation of the availability of a LAI-PrEP product, there is a need for rigorous behavioral and social science research to inform the development of tools to support decision-making around PrEP modalities that take into account the psychosocial factors identified in this study. Such factors—especially issues around the meaning of pill-taking for patients as an emotional burden versus a symbol of agency and control—will be critical to the development of patient education materials and shared decision-making processes with providers.
